# Enabling higher degree pathways for Aboriginal and Torres Strait Islander students

**DOI:** 10.1007/s13384-023-00626-8

**Published:** 2023-04-10

**Authors:** Shawana Andrews, Odette Mazel, Warwick Padgham

**Affiliations:** 1 Melbourne Poche Centre for Indigenous Health, Faculty of Medicine, Dentistry and Health Sciences, The University of Melbourne, 141 Barry Street, Carlton, VIC 3010 Australia; 2 Indigenous Law and Justice Hub, Melbourne Law School, The University of Melbourne, Carlton, VIC Australia

**Keywords:** Indigenous, PhD, Student support, Higher education, Research higher degree, Pathways, Social capital

## Abstract

Increasing the numbers of Indigenous people enrolled in research higher degrees in Australia is important for building the Indigenous academic workforce, broadening the scope of knowledge production in academic institutions and ensuring effective research outcomes for Indigenous Australians. While the numbers of Indigenous research higher degree students are increasing, universities still have a lot to do to bring that number up to parity. In this paper, we explore the value of a pre-doctoral program developed for Indigenous people interested in doing a PhD that provides them the information they need to inform their choices about undertaking a doctoral project. As the only program of this kind in Australia, this research contributes to the emerging literature on the factors that have an influence on why Indigenous people choose to undertake PhD programs and the effectiveness of initiatives to support their pathway to higher degree research. The research outcomes build on the evidence base for improving initiatives across the university sector, highlighting the need for tailored, Indigenous-led pre-doctoral support programs for Indigenous students, the value of cohort experiences and the importance of universities that value Indigenous people and their knowledge systems.

## Introduction

Increasing the numbers of Indigenous^1^ people undertaking higher degree by research (HDR) programs is important for strengthening Aboriginal and Torres Strait Islander leadership and workforce development (Ewen et al., 2019). Improved participation at this level also positively challenges and changes knowledge production in Australia’s major knowledge institutions and offers greater capacity for improving the health and wellbeing of Indigenous communities through experience-informed research initiatives (Bailey et al., 2020; Schofield et al., 2013). There has been significant growth in the numbers of Indigenous professionals graduating from undergraduate degree programs at universities across Australia (Bailey et al., 2020), however, underrepresentation of Indigenous people in research higher degrees remains significant (Behrendt et al., 2012; Trudgett, 2011, 2013; Trudgett et al., 2016). While a number of studies document the nature of this underrepresentation (Andersen et al., 2008; Behrendt et al., 2012; Bradley et al., 2008; Langton & Ma Rhea, 2009; Schofield et al., 2013; Whatman, 1995) the factors that have an influence on why Indigenous people choose to undertake HDR programs, and their experience through to graduation is less well researched (Barney, 2013, 2016; Meyer et al., 2020; Moreton-Robinson et al., 2020; Page et al., 2019; Trudgett, 2009, 2011, 2013). There is a critical need, therefore, to understand the factors that have an influence on access, participation, retention and completion for Indigenous HDR students, including the evaluation of existing initiatives, to better understand areas for improvement (Meyer et al., 2020).

This paper examines the effectiveness of an Indigenous-led, designed and delivered pre-doctoral program offered by the Melbourne Poche Centre for Indigenous Health at The University of Melbourne, for prospective Indigenous PhD students. While other universities offer year-long pre-doctoral fellowships for Indigenous students (such as RMIT), this is the only short course program of its kind in Australia that provides Indigenous people interested in doing a PhD the information they need to inform their choices about undertaking doctoral research. Through a semi-independent qualitative survey of past participants, our research highlights the factors that contribute to Indigenous people deciding to undertake a PhD and the impact that a targeted program can have on prospective Indigenous PhD students in their decision-making process. It shows the importance of Indigenous-led pre-doctoral support programs for Indigenous students, the value of cohort experiences and the significance of universities valuing Indigenous people, their knowledge systems and their intellectual pursuits.

## Literature: contextualising the need

In 2020, there were 586 Indigenous PhD students out of a total of 58,110 across all universities in Australia, representing 1% of the cohort (Australian Government Department of Education, Skills and Employment, 2022a, 2022b). While this is a significant increase (55%) from the numbers of Indigenous PhD students 10 years earlier (320 in 2010, making up 0.66% of the total student numbers) (Australian Government Department of Education, Skills and Employment, 2021), it still remains well short of the proportion needed for population parity.

In their paper on Indigenous higher education, Anderson, Bunda and Walter, called on universities to release the potential of Indigenous students through a coordinated and committed approach to transforming Indigenous pathways to and through higher education (Andersen et al., 2008). The key to success, they suggested in 2008, are programs that are responsive and tailored to Indigenous students’ needs and that are Indigenous-led. Important to this is a whole-of-university approach that employs Indigenous staff, values Indigenous-specific initiatives and encourages a sense of belonging (Andersen et al., 2008). Through her long history of research on Indigenous HDR students, Michelle Trudgett has expanded the knowledge on the factors that contribute to attracting, retaining and graduating Indigenous students. Her doctoral work, in line with Andersen et al., highlighted the need for tailored programs that account for the unique circumstance of Indigenous students including the role of Indigenous-specific centres, cohort experiences, quality supervision, adequate financial support and building family and community support (Trudgett, 2008, 2009).

Responding to the findings of the Bradley and Behrendt Reviews (Behrendt et al., 2012; Bradley et al., 2008), a number of government initiatives have, since that time, provided incentives for higher education institutions to increase their enrolments of Indigenous HDR students, including guaranteed stipends, weighted funding for Indigenous PhD completions and the development of networking opportunities (Indigenous Higher Education Advisory Council, 2011, 2016; *National Indigenous Research and Knowledges Network*, n.d.). Universities Australia also developed two strategies (2017–2020 and 2022–2025) to encourage commitment to, and implementation of, initiatives in this area. The latest strategy focusses on student and staff success, university responsibilities for Indigenous advancement, addressing racism and cultural safety in university environments, and valuing Indigenous knowledges (Universities Australia, 2017, 2022). The intentions of these initiatives, however, are yet to be fully realised and enrolment numbers, and more importantly completion numbers, remain too low (Gore et al., 2017). The messages of Indigenous-led and tailored programs, cohort experiences, and initiatives that affect the experience of belonging, such as Indigenous staff numbers and university values, remain at the heart of the solutions for the way forward (McKinley & Anderson, 2016; Moreton-Robinson et al., 2020; Page et al., 2019; Schofield et al., 2013; Trudgett, 2009, 2011, 2013). In a recent review of the literature on determinants for Indigenous participation in HDR, Hutchings et al. reiterate this message, but also call for more research on Indigenous HDR students’ experiences and the factors that have an impact on attraction to, and completion of, HDR studies (Hutchings et al., 2019, p. 247). Meyer et al. agree and emphasise the need to evaluate existing programs (Meyer et al., 2020). In this paper, we contribute to this call through an evaluation of the PhD Familiarisation Program delivered by the Melbourne Poche Centre for Indigenous Health that supports the enrolment of Indigenous PhD students across all Australian universities.

### Background: The Melbourne Poche PhD Familiarisation Program

The Melbourne Poche Centre for Indigenous Health was established in 2015 within the Faculty of Medicine Dentistry and Health Sciences (MDHS) at The University of Melbourne with philanthropic support from Greg Poche AO and Kay Van Norton Poche AO. It is part of a network of five Poche Centres in universities across Australia that each focus on a unique area of Indigenous health research and scholarship. The Melbourne Centre operates with two overarching strategic goals: (1) to build Indigenous health leadership; and (2) to create academic pathways. As part of the latter, the Centre undertakes a number of initiatives to facilitate Aboriginal and Torres Strait Islander peoples’ participation in doctoral research and to support their access, participation, retention and completion, including pre-enrolment and post-doctoral planning. This paper examines the Centre’s pre-enrolment initiative aimed to support increased PhD enrolments.

When the Centre was established, one of its targets was to recruit 20 new Indigenous PhD students to the Faculty of Medicine, Dentistry and Health Science (MDHS) by 2020. To achieve this, the Poche PhD Familiarisation Program was developed to provide pre-enrolment support for prospective students in a cohort setting and to introduce them to, or expand their knowledge on, some of the fundamentals about enrolling in and undertaking a PhD. The program aimed to fill an identified gap in Indigenous PhD student recruitment. Held annually, and in person, from 2015 to 2019 (before the disruption of COVID 19), the program was run over three days and involved a series of PhD preparation workshops providing the opportunity for people to network with Indigenous academic leaders, current PhD students and other prospective Indigenous PhD students. Participants were recruited through an expression of interest process aimed at those interested in pursuing a PhD within the next five years. Cohorts ranged in size from five to 13 participants and included multi-disciplinary and discipline-focussed groups. Participants were fully supported financially by the Centre to attend from all over Australia and while the Program was initially set up to recruit students to MDHS, it quickly evolved to include anyone interested in doing a PhD at any university.

The Familiarisation Program includes sessions on the purpose of undertaking a PhD, motivations and goals; the barriers to getting started; understanding the roles of supervisors and mentors; PhD application processes; the importance of formal and informal networks; and the opportunities and challenges of being an Indigenous research student within the academy. While these formed the foundation of each program iteration, workshops and introductory meetings were also included that were tailored to the specific cohort and individual participants. Participants were coached and supported to start, or further develop, a draft research question and proposal, and meetings with potential supervisors were facilitated, where possible. The program was Indigenous-designed and led, providing participants with a culturally safe space to discuss their research ideas, raise challenging issues and share their experiences. Many of the workshops drew in expert presenters and wherever possible, they were also Indigenous. The workshops were designed to encourage maximum engagement with supportive opportunities for open and honest dialogue. Social engagement across the program also deepened the cohort experience and provided opportunities for networking.

## Methods

The evaluation of the Familiarisation Program was Indigenous-led and utilised Indigenous methodologies to privilege an Indigenous worldview and voice (Rigney, 1999; Yunkaporta, 2019). The research was conducted from a baseline position that values Indigenous knowledge systems; recognises the roles of, and relationship between, the researchers and participants as influential parts of the research process; values reciprocity; and recognises lived experience as essential to developing knowledge (Kovach, 2009; Moreton-Robinson, 2013; Nakata, 2007a; Walter & Andersen, 2013). The project was conducted in line with the ethos set out by Anderson et al., namely, how universities should respond to, and act on, fulfilling the potential of Indigenous students (Andersen et al., 2008).

To better understand the experiences of prospective Indigenous PhD students and to evaluate the effectiveness of the Familiarisation Program, those who engaged in the Program between 2015 and 2019 were invited to participate in an online survey (Ethics ID Number: 20368). The survey was developed by the authors and was reviewed by an Indigenous PhD student and an Indigenous Professor external to the Centre prior to being distributed. Topics included:

- Family history in higher education
- Motivations for participating in the Program
- Factors that contribute to doing a PhD
- Which aspects of the Program were most beneficial and why, and
- How the Program could be improved.

The survey was conducted using Qualtrics technology and analysed thematically by the authors. It should be noted that Poche Centre Staff have no authority over PhD admissions processes.

## Results

### Poche PhD Familiarisation Participants who pursued higher research degrees

**Fig. 1:**
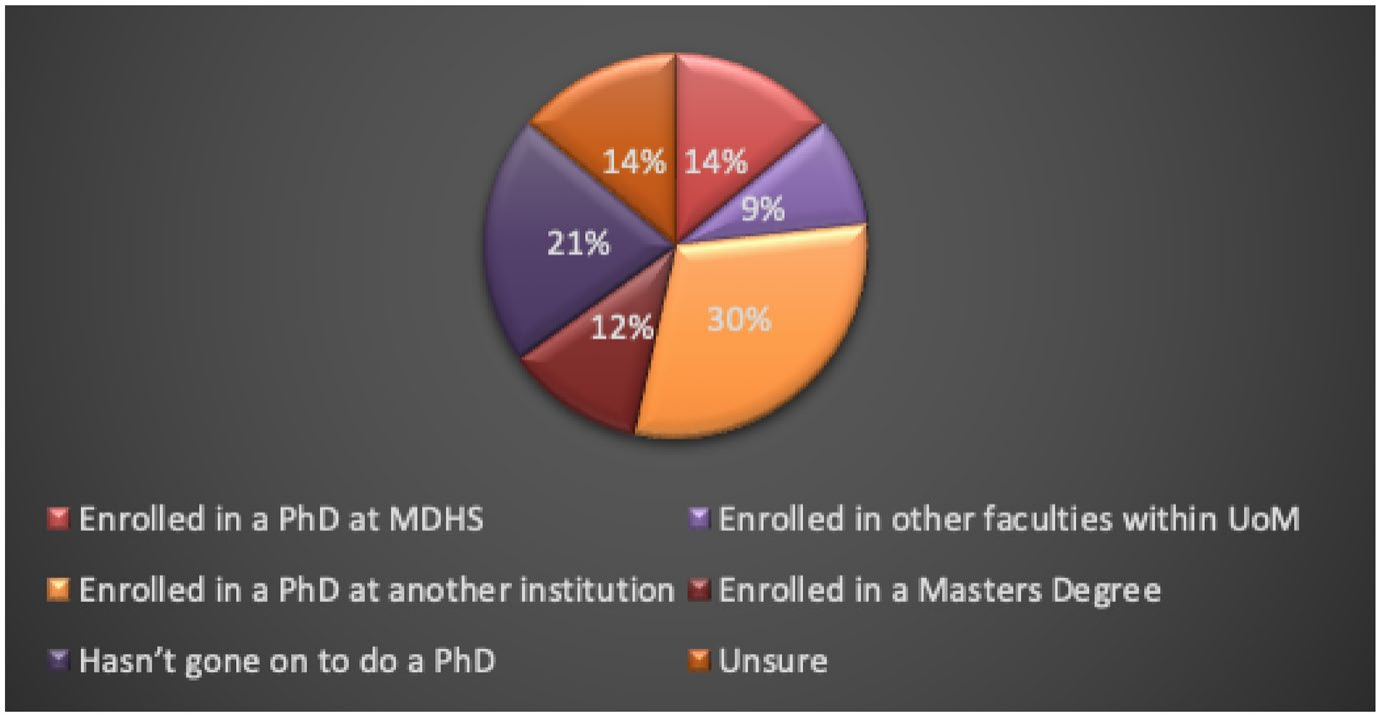
Number of PhD Familiarisation participants who enrolled in a PhD

From 2015 to 2019, 43 people participated in the Familiarisation Program. Of those, six (14%) enrolled in a PhD in MDHS, and another four (9%) in other faculties at The University of Melbourne. Thirteen (30%) were known by the researchers to have enrolled in a PhD at another institution, nine (21%) have not gone on to do a PhD and there are six (14%) whom we do not have further information about. While the program was not targeted at incentivising Master’s level enrolment, we know of five (12%) participants that undertook a Master’s Degree (see Fig. 1).

### Survey participant information

**Fig. 2:**
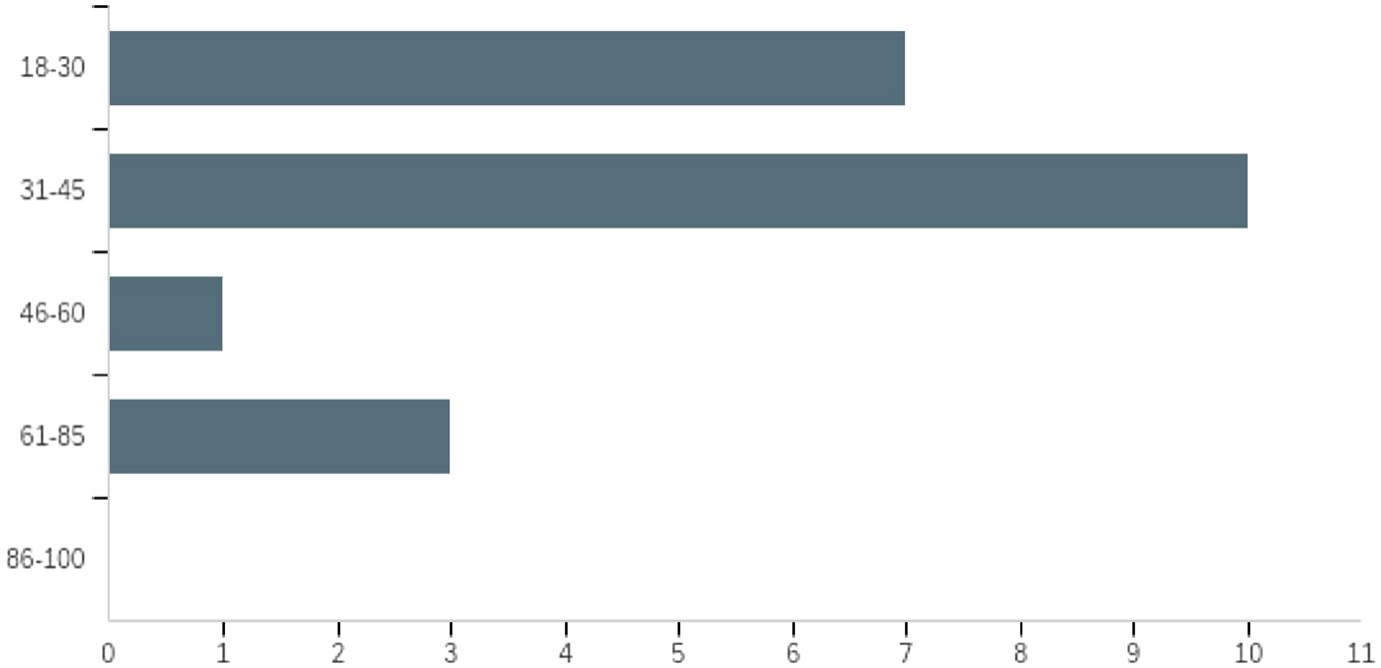
Age of participants who undertook the survey

Of the 43 Program participants, 21 (47%) completed the online survey. Eighteen identified as Aboriginal, two as Torres Strait Islander and one as both Aboriginal and Torres Strait Islander. Sixteen identified as female, four as male and one as gender non-binary. Most participants were between the ages of 31 and 45 (see Fig. 2).

Fifty-seven percent of the survey participants identified that they had caring responsibilities that included children, parents and/or other family/kinship. Eleven participants (52%) had other members of their immediate family who had completed tertiary studies. Of those family members, the majority (82%) had completed a Bachelor’s degree, one had a Master’s degree and only one family member had completed a PhD.

### Motivation for doing the Program

Survey participants were made aware of the PhD Familiarisation Program in a range of ways, including through recommendations from a friend or colleague (44%), via social media (13%), the Centre’s Newsletter (12%), other media (12%) or were approached directly via email (18%). These results highlight the strength of word of mouth as well as social connections, with one survey participant saying that they enrolled because they ‘had seen first-hand the success of the program and what past attendees had gone on to achieve’. All participants had an interest in undertaking a PhD, but some were further along the path towards enrolling than others. Participants expressed motivations for undertaking the Familiarisation Program that included: better understanding the steps involved in applying for a PhD; being informed about the opportunities available for Indigenous students; and having a better grasp of what it really means to do a doctoral project:

I have been considering if I should try to apply for a PhD, but I wasn't sure what a PhD involves and how to apply.

Some noted the ‘opportunity to network and learn more’ with others in the same position as them, as well as with academics across the university.

### Program design

#### Indigenous-led

Survey participants noted the importance for them that the program was Indigenous-designed and led. For many it was integral to them deciding to enrol:

I wouldn't have considered taking part if it wasn't Indigenous. I knew that an Indigenous program would meet my needs as an Aboriginal person, give me the support I required, and address my thinking around a topic and area of study.

**Fig. 3:**
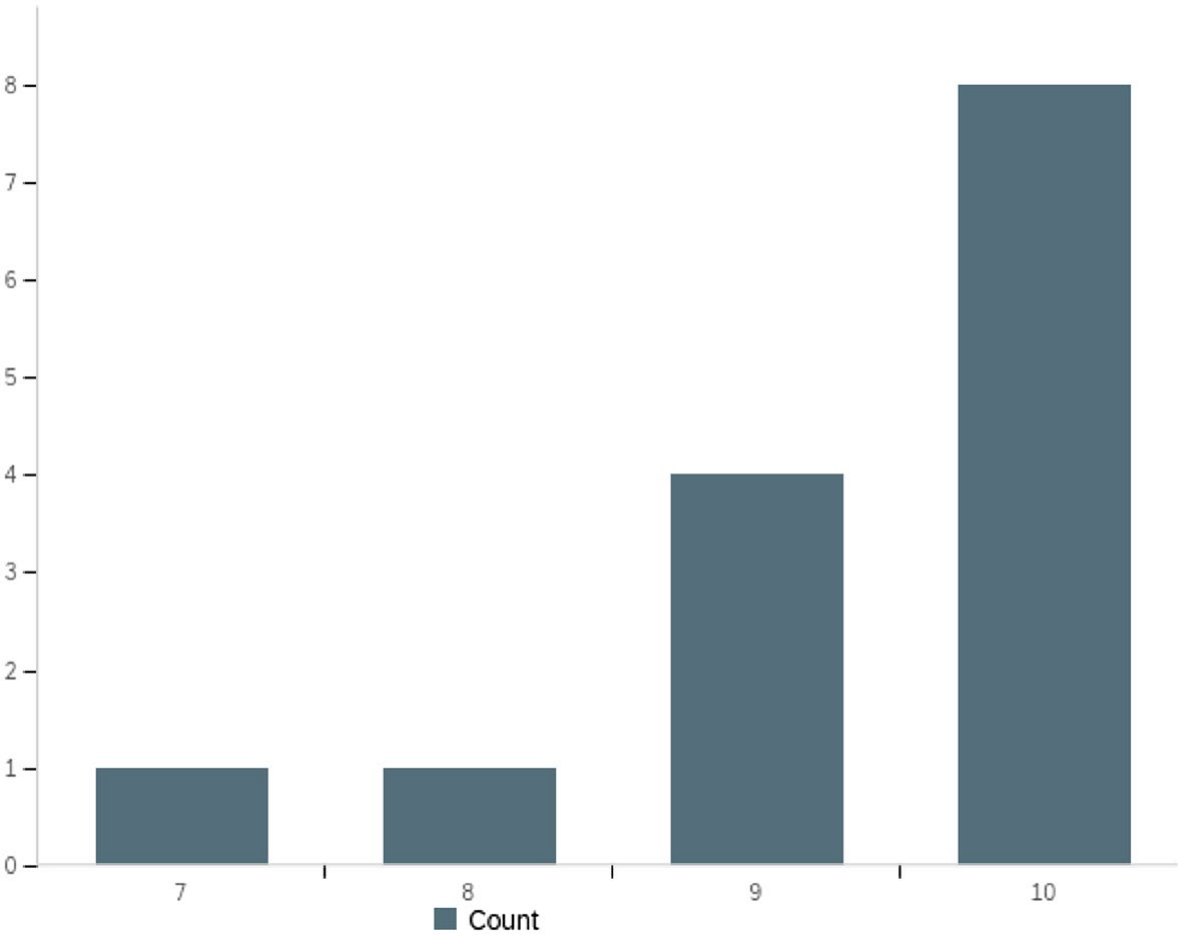
Value of Indigenous-led Program on a scale of 1–10

Participants were asked on a scale of 1–10 how important it was to them that the program was Indigenous-led (with 10 being the most important) and all responses, shown in Fig. 3, ranged between 7 and 10, with a mean of 9.36, showing that this was a significant factor in their experience of the program.

When asked to explain their responses, participants said they felt the program took into account their lived experiences and personal trajectories:

Having programs founded in Indigenous knowledges (knowing, being, doing) with the personal lived narrative and understanding – it is important. Our journey and the fact we are working in colonised systems, is different to other Australian PhD students.

I felt that it was tailored to our view and expectations and took into account the lack of confidence I experienced.

Many noted that it made the experience culturally safe and that they felt more comfortable engaging with the content and asking questions:

Without this being led by community I would not have attended and never headed down the path I did. It was culturally safe and when entering an institution that’s traditionally been designed to discriminate and traumatise my people it was absolutely essential that this program was designed, led and run by Aboriginal community.

Culturally safe spaces. Feeling 'equal' to the other participants. Balance of power between facilitators and participants.

#### Tailored to the individual

The participants surveyed noted the benefits of a tailored program and a small group size that meant each person was afforded the time and attention they needed: ‘The program was fantastic. Discussions were tailored to each participant. The team was very supportive. The small size of group also was also great’. Many reported that the Program gave them a good understanding of the university environment; what to expect with regards to the challenges as well as highlights of doing a PhD; as well as what financial and other supports were available to them as a student:

It inspired and motivated me. It also gave me an understanding of what a PhD is, options for potential projects (applying for a project that is already set up or pursing our own research question), and how to apply.

We often hear the PhD journey is a lonely journey but throughout the program it was made evident that with the right supports and outlets the process is manageable.

Opportunities to network and talk with Indigenous academics, as well as potential supervisors were identified as important components of the Program. Survey participants expressed how useful it was to hear about Indigenous academics’ journeys and their experience in the academy. For some, the connections made at that time have led to ongoing relationships:

It was really important for me to hear from other Indigenous academics what their PhD experiences were like and the career opportunities they had since their PhD. This gave me an honest insight into what the academy would be like for me as an Aboriginal person.

It opened up networks, introduced me to supervisors, and other academic personnel that I am still in contact with today.

Linking participants with potential supervisors assisted many in taking the next steps along the application process. It provided the opportunity to find supervisors that might be a good fit and contributed to people getting a clearer idea of what the focus of their research might be: ‘It helped me clarify…and articulate my area of research focus [through] the exhausting process of speed dating with potential schools, and supervisors’.

#### Cohort experience

Survey participants overwhelmingly commented on the impact of the cohort experience and the benefit of undertaking the program with other Indigenous people with whom they had many shared experiences, both in terms of life generally, but also with regard to their interests and career trajectories. Many felt that being part of a cohort assisted them in working through the idea and reality of doing a PhD, and provided them the opportunity to air their anxieties, discuss the potentials and work through the processes with people who understood where they were coming from:

It meant that I would be in the room with other Aboriginal people thinking along the same lines as myself; talking about similar ideas and issues that I was contemplating; and understanding each other and being able to converse easily.

Again, it added to feelings of cultural safety and being understood, contributing somewhat to making the university environment more welcoming and inclusive.

People drew inspiration and courage from the cohort and for some, the shared endeavour was pivotal for them deciding to enrol in a PhD:

It was very comfortable being a part of a group of people like me who were aiming high. It made questions more real, and anxiety more valid, and our hope more authentic somehow – being in this together.

I drew inspiration from the other participants hearing about their stories of resilience and success. They were like-minded people that have become friends and supporters.

**Fig. 4:**
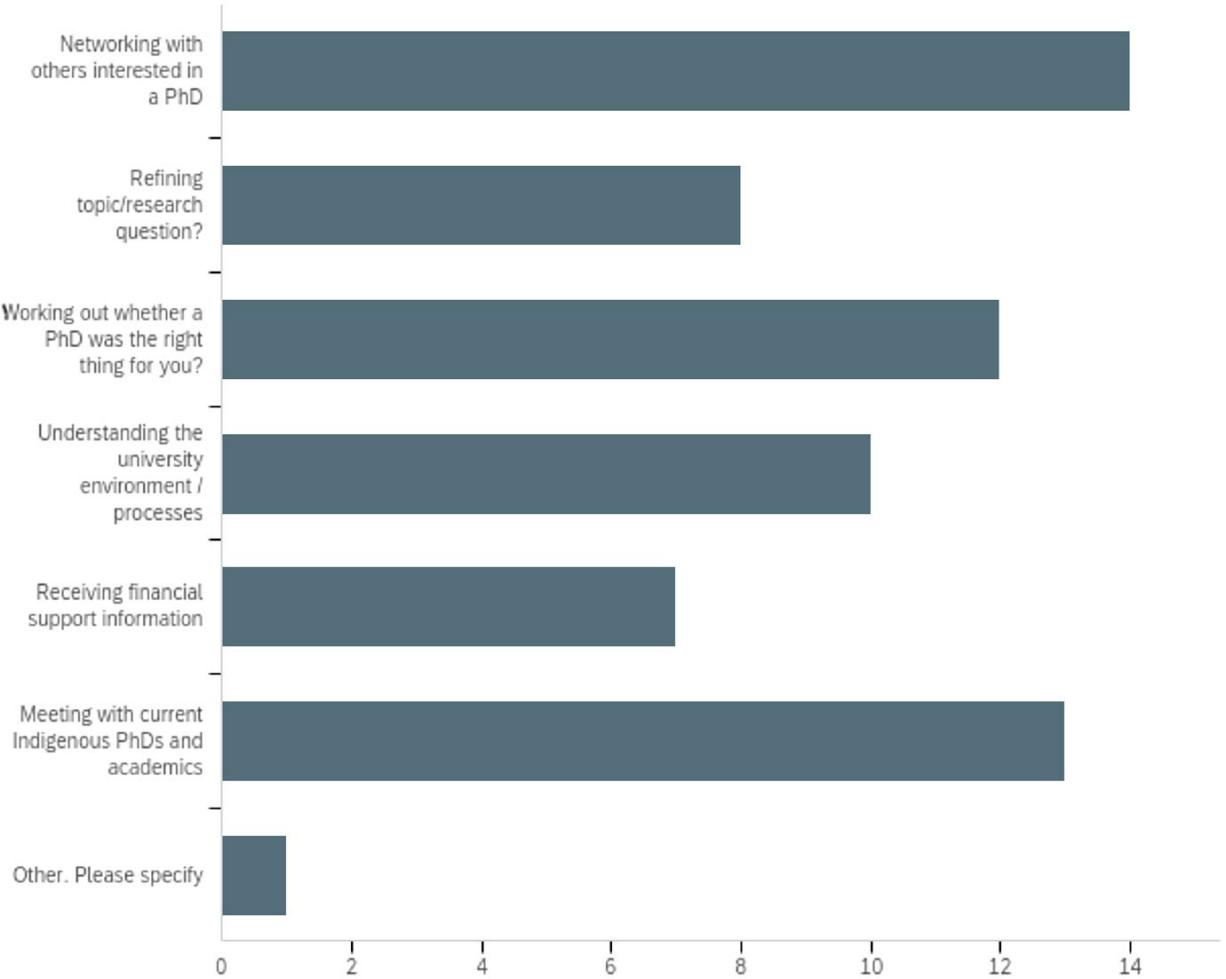
Most beneficial aspects of the Familiarisation Program

While some participants knew one another, others made new connections. Many of these connections extended beyond the time of the Program (Fig. 4).

### Impact of the program

Survey participants reported that undertaking the Program had a positive impact on them, both personally and professionally, commenting that it gave them the information necessary and provided them the confidence and courage to undertake a PhD:

It completely made me feel empowered to enrol, that I could do this even though none of my family have done any formal education. I felt there were some really great supports available to be successful and help me in any areas I may struggle in. And, most importantly I felt a sense of community, belonging and that I was welcomed.

Personally, it gave me confidence that I needed to start on this journey, I was good enough, I was more than capable in doing this.

For others it clarified that it was not the right time for them to do a PhD:

At a professional level it assisted me in making a huge decision, and I realised that it wasn't the right time for me to take up study and that I needed to continue with my employment for the time being.

Overall, participants reported the benefits of getting an insight into the realities of doing a PhD, better understanding university processes, and being introduced to other Indigenous people who had done a PhD, were currently enrolled, or who were thinking about it. This all contributed to them being able to envisage what doing a PhD might be like and having the tools to proceed with the process if they chose to.

Before the program I was really on the fence about doing a PhD and the program was the tipping point for me in realising that a PhD was right for me and giving me a good understanding of what the university environment would really be like. Being able to meet current Indigenous PhD students and academics helped me gain a better idea of what a PhD would realistically involve and having an opportunity to meet other interested PhD students helped me gain supportive connections that I have maintained throughout my PhD.

### Program improvements

The program, which has run five times, has evolved over that time with feedback from participants following each delivery. Small but important improvements such as having discipline-specific program iterations, more time with current PhD students and increased networking and social engagement, have been made to ensure the program is responsive to participant experiences.

Those who undertook the current survey also provided useful perspectives on how the program can be improved. Having had time to reflect on the program, and with some having completed their PhD, their insights are invaluable. Overall, participants suggested that more unstructured time to engage and network with other participants as well as Faculty academics and professional staff would be useful. They were also interested in having more time to talk through how to choose the right supervisor, how to navigate the potential pitfalls of supervision, and how, if necessary, to change supervisors. Participants noted that even more time with current PhD students would be useful including more in-depth and diverse discussions about the challenges as well as successes along the PhD journey. It was suggested that this could be usefully paired with information on self-care, milestone moments, and ways to deal with and manage racism in the university environment.

In terms of application processes, participants suggested that tips or techniques for ‘selling yourself’ would be useful, including ways that they can present their lived experience and grassroots work as valuable in their PhD applications. This also points to the need to focus more on ways to frame Indigenous methodological and research approaches and protocols as valuable and necessary. Survey participants would have liked more discussion on career pathways other than academia following the PhD, and several participants commented that it would be good to have a formal follow-up process and a point of contact or mentor to help them work through their PhD application further. People also suggested that it would be beneficial to re-connect with fellow participants down the track to check in and see where everyone landed: ‘It would have been interesting to hear what others from the cohort were doing…’.

### University systems improvements

We asked survey participants about what makes one university more appealing than another when looking to do a PhD. Many respondents noted the importance of global reputations and the value of attending a Group of Eight university. Others said it was dependent on the right supervisors, the financial and other support systems available, the location, or the option to study remotely. Many noted the importance of a strong Indigenous presence or visibility on campus along with considerations of the track record of the university’s engagement with Indigenous people and local communities, as well as valuing Indigenous knowledges.

As an Aboriginal researcher I think universities that have good reputations and engagement with Aboriginal and Torres Strait Islander communities are most appealing.

With respect to attracting more Indigenous PhD students, participants cited the need for universities to better engage Indigenous students with the prospect of undertaking doctoral studies during their undergraduate years and to improve aspirational messaging: ‘raise student awareness of the benefits of PhD study in terms of their future employment and financial and the benefits of research for Aboriginal peoples and communities generally’. They also flagged the need for universities to provide non-traditional pathways into a PhD ‘…we all come from different walks of life, non-traditional pathways into PhD for Aboriginal and Torres Strait Islander students are needed’. Participants also noted the importance of having properly trained PhD supervisors in Indigenous methodologies and culturally safe practices. Personal emails targeting individuals who might likely be interested were also seen as a way of getting people interested—providing a strong message that the university is interested in them.

## Discussion

### Belonging in the university environment

Universities have, historically, been critical contributors to colonial practices, producing ‘knowledge’ that supported the control and management of Indigenous communities (Goldberg, 1993; Lovett et al., 2020; Moreton-Robinson, 2011; Nakata, 2007b; Rigney, 2001; Walter & Butler, 2013). There have been a number of initiatives over the last 30 years aimed at shifting this legacy and re-orienting the value attributed to Indigenous peoples and knowledge systems, but there remains significant work to ensure that universities are culturally appropriate, safe and inclusive places for Indigenous people (Bailey, 2016; Carter et al., 2018; Taylor et al., 2019). This racist history along with the systemic impact of discrimination across almost all areas of life have meant Indigenous people have been excluded from higher education and, in particular, HDR programs (Bodkin-Andrews & Carlson, 2016; Brown, 2019; Gray & Beresford, 2008). Most Indigenous students currently undertaking a PhD are the first in their family, and the first in their community, to embark on a HDR project. This has a significant impact on experiences of belonging, system navigation and confidence, and reduces the scope of a student’s network of support. Universities, therefore, have a responsibility to fund and deliver initiatives that address the embedded systemic barriers and respond to the nuances of Indigenous engagement with higher education.

The Familiarisation Program orients prospective PhD students to the HDR environment. Specifically, it interrogates the nature of being an Indigenous PhD student and provides the building blocks for navigating the university system, with a view to establishing confidence as a foundation for a sense of belonging—a noted issue for Indigenous students along the education continuum (Bodkin-Andrews et al., 2010). Importantly, the Program takes into consideration prospective students’ lived experience and responds to individual circumstances and complexities with tailored advice, support and networks. Attending to theses nuances offers a highly relational level of engagement that is consistent with Indigenous ways of knowing, being and doing, but unusual in the higher education context (Hogarth, 2022). As an Indigenous-led initiative within a colonial institution, it carves a space within the university environment that is culturally safe and responsive to Indigenous people and is a signifier of the value of the intellectual contribution of Indigenous people through higher degree research.

Essential to experiences of belonging are seeing and engaging with other Indigenous staff and students in the university sector (Buckskin et al., 2018). For those who undertake the Program, having access to Indigenous academics as well as current PhD students, shifts the sense of dislocation and provides important elements of recognition and relationality as well as examples of excellence and success (Martin, 2003). Further, being exposed to the work that is occurring within the institution—work that focusses on Indigenous issues, utilises Indigenous methodologies and shifts the nature of critique and knowledge production to incorporate Indigenous ways of knowing, being and doing—is empowering and provides access to intellectual growth and academic success.

### Building social capital: Indigenous programs for Indigenous people

Building on the work of Bourdieu, Coleman and Woolcock (Bourdieu, 1984; Coleman, 1988; Woolcock, 1998), Maggie Walter conceptualises Indigenous social capital as ‘networks of social relationships that provide mutual benefit in terms of reciprocity, trust, shared values and norms, information and cooperation’ (Walter, 2015, p. 71). She argues that it is essential for Indigenous people to build social capital within spaces such as higher education institutions (Walter, 2015, pp. 82–83). As an Indigenous-designed, led and delivered program, the Familiarisation Program improves accessibility and ensures that the academic space is seen and experienced as a rightful place for Indigenous people (). By bringing together people interested in realising their ambition within the academy, it creates the opportunity to develop connections with others in a similar position and with similar aspirations. As Trudgett and Andersen et al. identified (Andersen et al., 2008; Trudgett, 2008, 2009), there is value in having a critical mass, or a cohort experience to reduce feelings of isolation and marginalisation and to develop beneficial engagements built on trust, shared values and reciprocity. The cohort nature of the Program creates a potent environment in which Indigenous identities and knowledges are affirmed and prioritised (Brown, 2010), and a space within which social capital is built.

### Pathways forward

Attracting Indigenous PhD students is only one part of a long pathway in and through higher education for Indigenous people. Activities along that continuum must be coordinated and continuous, providing Indigenous people the aspirations and pathways to, through and beyond higher education (Barney, 2016; Dillon et al., 2020; Frawley et al., 2017; Nobin et al., 2013). Studies show that Indigenous researchers contribute to improved services and outcomes for Indigenous people, but they will also have an impact, as we have shown, in recruiting and graduating more Indigenous students (Ewen et al., 2019; Page et al., 2019; Trudgett, 2009). While initiatives within higher education institutions have improved significantly and the number of Indigenous people in doctoral programs is rising, there is still much that universities can do to ensure they are places that Indigenous people want to invest in and commit to.

The PhD Familiarisation Program is the only program of its kind across Australia. While its philanthropic foundation allows for some program flexibility and creativity, we note that initiatives that are aimed at recruiting and supporting Indigenous students in the sector should be regarded as core business and funded accordingly in all higher education institutions across Australia. Claiming space and navigating the political and economic nuances in the academy is complex. In areas of Indigenous intellectual and academic development the barriers are particularly pronounced. The siloed and disconnected educational pathways of Indigenous students require further oversight and integrated opportunities.

## Conclusion

This research shows that what universities need to do to graduate more Indigenous PhD students remains much the same. Providing tailored, Indigenous-led programs through cohort experiences within a university that clearly values Indigenous people and knowledges will have an impact on Indigenous people considering a PhD. Universities have much to gain from supporting Indigenous academic and intellectual development, embracing Indigenous methodologies, and championing Indigenous values (Larkin, 2011; Moodie et al., 2018; Nakata & Maddison, 2019). In fact, as the centres of knowledge production in Australia, it is their obligation. Building the Indigenous academic workforce will not only have an impact on those individuals, their families and communities, but will increase the numbers of Indigenous PhD students into the future, challenge and change knowledge production in the academy, and improve services for Indigenous people (Bailey et al., 2020; Schofield et al., 2013). Educational aspiration is present in every Indigenous community in Australia. Doctoral level education is part of this aspiration, universities just need to provide the appropriate environment necessary to support the priorities, values and pedagogical needs of Indigenous peoples.

This research is limited in its scope as a single-site evaluation because it is the only program of its kind in Australia. It is important, however, to highlight, through such evaluation and research, the value of Indigenous-specific programs and support initiatives in higher education to make the case (even more loudly) for core university funding, support and systems change. This research also reinforces the need for further investigation of alternative pathways to a PhD (Arnold, 2017), and data collection on the journey through and beyond academia for Indigenous PhD students (Locke et al., 2021; Povey et al., 2022).

## Footnotes

1 We use the term ‘Indigenous’ to inclusively refer to Aboriginal and Torres Strait Islander people of Australia.
